# Balance billing: the patients' perspective

**DOI:** 10.1186/2191-1991-1-14

**Published:** 2011-09-17

**Authors:** Mathias Kifmann, Florian Scheuer

**Affiliations:** 1 Universität Hamburg, Fakultät Wirtschafts-und Sozialwissenschaften, Von-Melle-Park 5, 20146 Hamburg, Germany; 2 Stanford University, Department of Economics, Stanford, CA 94305, USA

**Keywords:** physician reimbursement, price controls, Medicare

## Abstract

We study the effects of 'balance billing', i.e., allowing physicians to charge a fee from patients in addition to the fee paid by Medicare. First, we show that on pure efficiency grounds the optimal Medicare fee under balance billing is zero. An active Medicare policy thus can only be justified when distributional concerns are accounted for. Extending the analysis by Glazer and McGuire, we therefore analyze the optimal policy from the patients' point of view. We demonstrate that, from the patients' perspective, a positive fee can be superior under balance billing. Furthermore, patient welfare can be lower if balance billing is prohibited. In particular, this is the case if the administrative costs of Medicare are large. However, we cannot rule out that prohibiting balance billing may be superior. Finally, we show that payer fee discrimination increases patient welfare if Medicare's administrative costs are high or if Medicare's optimal fee under balance billing implies lower quality for fee-only patients.

*JEL***-classification: **I11, I18, H51

## 1 Introduction

The US Medicare program allows doctors to 'balance bill' patients, i.e., to charge them a price in addition to the Medicare payment. In the late 80s and early 90s, state and federal legislation was introduced to restrict this practice. Additional prices are now limited to about 10% of the Medicare fee.(endnote a) In a theoretical study, Glazer and McGuire have shown that these restrictions on balance billing come at a price as doctors have an incentive to reduce the quality of their services [1]. Strikingly, prohibiting balance billing reduces quality for all patients, regardless of whether they pay a balance bill. From an efficiency point of view, they demonstrate that allowing balance billing always leads to superior results if the Medicare fee is set appropriately.

A limitation of the analysis by Glazer and McGuire is that they focus exclusively on the efficiency aspects of balance billing. An important concern, however, is that patients are worse off if physicians are allowed to balance bill. In particular, previous work by Paringer, Mitchell and Cromwell as well as Zuckerman and Holahan has shown that allowing physicians to charge extra fees may only increase the rents of physicians at the expense of patients [2-4]. These papers, however, do not consider effects on quality. Taking into account efficiency gains from balance billing, this raises the question on how these gains are shared between patients and physicians.

In this paper, we take the analysis of Glazer and McGuire further and focus on the welfare of patients. We analyze the optimal Medicare fee both from a pure efficiency perspective and from the patients' point of view. Furthermore, we reexamine the case for prohibiting balance billing and consider the effects on patient welfare if Medicare discriminates the fee depending on whether the physician treats the patient at the fee only or charges a balance bill.

The paper proceeds as follows. In Section 2, we discuss the literature. Section 3 reviews the analysis by Glazer and McGuire. In Section 4, we determine the optimal Medicare fee under balance billing using the social surplus function of Glazer and McGuire. Section 5 analyzes the implications of Medicare's policy on patient welfare. Section 6 concludes the paper.

## 2 Review of the literature

Most of the theoretical studies on balance billing assume a monopolistic physician who faces a downward-sloping demand curve [2]-[4]. Within this framework, the effects on the quantity of services supplied by the physician has been explored. The physician is able to price discriminate, requiring patients with a high willingness to pay a balance bill. If the physician also accepts fee-only patients under balance billing, then prohibiting balance billing leaves the quantity of supply unchanged since only inframarginal patients are balance billed. Only the physician's rent is reduced. However, if doctors refuse to treat fee-only patients under balance billing, then prohibiting balance billing reduces the number of patients treated.

How Medicare's balance billing policy affects the incentives for a monopolistic physician to set quality of treatment is analyzed by Feldman and Sloan as well as Wedig, Mitchell and Cromwell [5,6]. Both papers assume that the physician is not able to price or quality discriminate. Feldman and Sloan show that it is uncertain whether price controls, i.e., prohibiting balance billing, increase welfare. Wedig et al., however, find a case for price controls if health insurance shifts the demand curve to the right and physicians react by increasing quantity and quality beyond the social optimum.

All the models presented do not include competition among physicians. Furthermore, neither Feldman and Sloan nor Wedig et al. consider price and quality discrimination. However, these factors are highly relevant in the context of balance billing. First, Medicare's fee policy affects the degree of competition between physicians. Second, balance billed patients are likely to receive higher quality than fee-only patients. Both factors are incorporated in the model by Glazer and McGuire. They show that physicians have an incentive to save costs by reducing quality for Medicare patients. To patients who pay a balance bill, however, they will provide the efficient quality level. Their main result is that by setting fees correctly, efficiency is higher if balance billing is allowed.

An empirical study of the effects of Medicare restrictions on balance billing in late 80s and early 90s has been performed by McKnight [7]. She finds that these reduced out-of-pocket medical expenditure of Medicare beneficiaries by 9%. With the exception of a significant fall in the number of follow-up telephone calls, her study shows little evidence that physicians changed their behavior in response to the balance billing restrictions.

## 3 The analysis by Glazer and McGuire

### 3.1 The model

In the model by Glazer and McGuire, patients demand one unit of service per period and are uniformly distributed on a line segment of length one. The two physicians are situated at the end points of this segment. A patient's distance from a physician captures the product differentiation which implies that each supplier faces a downward-sloping demand curve. It serves as a geographic metaphor for patients' preferences for treatment.

Demand results endogenously from the benefit Ū-t-s that a patient with distance *t *from the physician (0 ≤ *t *≤ 1) derives from a service of quality *s*, where higher values of *s *indicate a lower level of quality. Medicare sets the fee *f *for a unit of service. In addition, physicians may ask certain patients to pay a price *p*.(endnote b) Assuming symmetric information about the patients' willingness to pay, physicians will ask the price *p *from the patients situated close to them (as they have a high willingness to pay) and renounce on it for the others. This market segmentation is enforceable as physician services cannot be traded. Physicians also choose the quality offered to price-paying and fee-only patients. Apart from affecting demand, quality increases costs. For a given quality, marginal cost is constant and equals *c *for *s *= 0. Quality reductions result in positive but diminishing cost savings *v*(*s*) with *v*(0) = 0, *v*' *>*0 and *v*″ *<*0.

Glazer and McGuire show that a profit maximizing physician always sets the service quality for the price-paying patients on the efficient level determined by *v*'(s*) = 1. This is due to the additive specification of the utility U(p,t,s)=Ū-p-t-s that a price-paying patient with distance *t *from the physician draws from a service of quality *s*. A one unit increase in the price has the same impact on the patient as a quality reduction (increase of *s*) by one unit. The physician's profit from serving a patient is π(*p*, *s*) = *p *+ *f *- *c *+ *v*(*s*). For *v*'(*s*) *>*1, the physician can thus decrease the price by one and in return increase *s *by a unit. While the patient's utility is unaffected, her profit rises. An analogous argument rules out quality levels *s *with *v*'(*s*) *<*1. Because social surplus from a unit of service U(p,t,s)+π(p,s)-f=Ū-t-s-c+v(s) is maximal if quality is such that *v*'(s*) = 1, price-paying patients always receive the socially efficient level of quality, independently from Medicare's fee policy *f*. For this reason s* can be normalized to 0 and we have *v*'(0) = 1. *s *then measures the quality difference to the fee-only patients.

### 3.2 Market equilibrium

With the above assumptions, the physicians' demand functions can be derived. Let $t_{i}^{*}$ denote the total number of patients served by physician *i*. For each of them, she is paid *f *by Medicare. Patients with a high willingness to pay due to their short distance (and their long distance from the competitor) are asked to pay the price *p_i _*in addition. Their number is denoted by ${\tilde{t}}_{i}$. They receive the constant quality *s *= 0, whereas *s_i _*is the quality offered by *i *to her fee-only patients.

When discriminating patients, physicians' are limited by their patients' option to go to the other physician. They will only be willing to pay *p_i _*if this is superior to seeking treatment from the other physician with quality *s_j _*at the fee only. For the indifferent price-paying patient with distance ${\tilde{t}}_{i}$ from physician *i *the equality $Ū-{\tilde{t}}_{i}-p_{i}=Ū-\left(1-{\tilde{t}}_{i}\right)-s_{j}$ must hold.(endnote c) The number of patients from which physician *i *asks to pay *p_i _*is thus given by

(1) $${\tilde{t}}_{i}=\frac{1-p_{i}+s_{j}}{2}.$$

This shows that the discrimination rule of physicians is based on an endogenous limit between fee-only and balance-billed patients.

Analogously, the total number of *i*'s patients follows from the indifference $Ū-t_{i}^{*}-s_{i}=Ū-\left(1-t_{i}^{*}\right)-s_{j}$ and is

(2) $$t_{i}^{*}=\frac{1-s_{i}+s_{j}}{2}.$$

Thus, the number of *i*'s fee-only patients amounts to

(3) $${\hat{t}}_{i}=t_{i}^{*}-{\tilde{t}}_{i}=\frac{p_{i}-s_{i}}{2}.$$

Using (1) and (3), physician *i*'s profit can be written as a function of her strategy vector (*p_i_*, *s_i_*) and of her competitor's one:

$$\begin{array}{ll} \pi^{i}\left(p_{i},s_{i},s_{j}\right) & =\left(p_{i}+f-c\right){\tilde{t}}_{i}+\left(f-c+v\left(s_{i}\right)\right){\hat{t}}_{i}\;\\ & =\frac{p_{i}\left(1-p_{i}+s_{j}\right)}{2}+\left(f-c\right)\frac{1-s_{i}+s_{j}}{2}+v\left(s_{i}\right)\frac{p_{i}-s_{i}}{2}.\;\\ & \; \end{array}$$

The market equilibrium is a Nash equilibrium of the complete information game where

1. the two physicians simultaneously choose the price *p *for the price-paying and the quality *s *for the fee-only patients, and

2. each patient chooses one physician.

This market equilibrium yields an endogenous number of balanced-billed patients $\left({\tilde{t}}_{1}+{\tilde{t}}_{2}=\left(1-p_{1}-p_{2}+s_{1}+s_{2}\right)∕2\right)$ and fee-only patients $\left(1-{\tilde{t}}_{1}-{\tilde{t}}_{2}\right)$.

The necessary conditions for such an equilibrium are given by

(4) $$\partial \pi^{i}∕\partial p_{i}=\left[1-2p_{i}+s_{j}+v\left(s_{i}\right)\right]∕2=0$$

(5) $$\partial \pi^{i}∕\partial s_{i}=\left[-\left(f-c\right)+v^{\prime }\left(s_{i}\right)\left(p_{i}-s_{i}\right)-v\left(s_{i}\right)\right]∕2=0.$$

**Figure 1:**
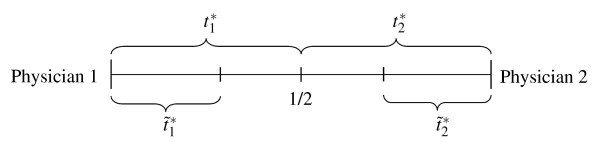
**Symmetric equilibrium in the market for physician services**.

Assuming that the second-order conditions are met, Glazer and McGuire confine their analysis to symmetric and stable equilibria (see Figure 1), whereby stability requires the slope of the reaction functions in the equilibrium to be smaller than one. This implies for *s_i _*= *s_j _*= *s *and *p_i _*= *p_j _*= *p*

(6) $$\partial s_{i}∕\partial s_{j}<1\Leftrightarrow v^{\prime \prime }\left(s\right)\left(1-s+v\left(s\right)\right)-3v^{\prime }\left(s\right)+{\left(v^{\prime }\left(s\right)\right)}^{2}<0.\left(\text{endnote d}\right)$$

Based on this condition, it is possible to show that in the symmetric Nash equilibrium quality increases with the fee *f*. Accounting for symmetry, (4) and (5) can be rewritten as

(7) 1-2p+s+v(s)=0

(8) $$-\left(f-c\right)+v^{\prime }\left(s\right)\left(p-s\right)-v\left(s\right)=0.$$

Substituting *p *= (1 + *s *+ *v*(*s*))/2 from (7) in (8) yields

(9) $$v^{\prime }\left(s\right)\left(1+v\left(s\right)-s\right)-2v\left(s\right)=2\left(f-c\right).$$

By implicit differentiation of (9),

(10) $$\frac{\text{d}s}{\text{d}f}=\frac{2}{v^{\prime \prime }\left(s\right)\left(1-s+v\left(s\right)\right)-3v^{\prime }\left(s\right)+{\left(v^{\prime }\left(s\right)\right)}^{2}}<0$$

because of (6). Physicians compensate for the lower fee by cost savings from reduced quality for the fee-only patients. Furthermore note that (7) implies

$$\frac{\text{d}p}{\text{d}f}=\frac{s+v^{\prime }\left(s\right)}{2}\frac{\text{d}s}{\text{d}f}<0$$

i.e., physicians reduce the price for higher quality if Medicare raises its fee.

### 3.3 Welfare analysis based on efficiency

In the symmetric market equilibrium, both the quality *s *for the fee-only patients and the price *p *for the price-payers depend on the fee *f *(cf. conditions (7) and (8)). The model can therefore be used to characterize the socially optimal fee *f^p ^*if price and quality discrimination are allowed. Because patients split evenly between physicians in the symmetric equilibrium independently from *f*, the preference parameter *t *is not relevant for this analysis. Glazer and McGuire consider a social surplus function where the price *p *as a pure transfer from patients to physicians does not enter. Concerning efficiency, only quality hence remains crucial and total welfare with price discrimination can be written as

(11) $$W^{p}=\int_{0}^{1}\left[v\left(s\left(t\right)\right)-s\left(t\right)\right]\text{d}t-\theta f.$$

Here, *v*(*s*) - *s *measures the net social gain of quality *s *per unit of service. θ*f *corresponds to the social cost of Medicare, where θ *>*0 indicates positive administrative costs.

The net social gain vanishes for *s *= 0 and is negative for all other values of *s *due to *v*(0) = 0, *v*'(0) = 1 and the concavity of *v*. In a first-best world, the fee *f *would therefore be chosen such that *s *= 0 for all patients. This level is denoted by f*. By equation (9), we obtain f* = 1/2 + *c*.

With positive social cost θ*f*, Glazer and McGuire identify a trade-off between quality and distortion costs if Medicare cannot dictate the level of quality and set *s *= 0 and *f *= 0 at the same time. Using equation (10), they show that the second-best optimal fee is f^p ^< f*, implying *s*(*f^p^*) *>*0, i.e., the second-best quality received by the fee-only patients is lower than that of the price-paying patients.

Glazer and McGuire compare the welfare level under price and quality discrimination and fee policy *f^p ^*with the situation where discrimination is prohibited. As shown in the preceding section, total demand of physician *i *then consists only of fee-only patients whose number is $t_{i}^{*}=\left(1-s_{i}+s_{j}\right)∕2$. Consequently, profit is π*^i^*(*s_i_*, *s_j_*) = (*f *- *c *+ *v*(*s_i_*))(1 - *s_i _*+ *s_j_*)/2 with the first-order condition for a maximum dπ*^i^*/d*s_i _*= *v*'(*s_i_*)(1 + *s_j _*- *s_i_*)/2 - (*f *- *c *+ *v*(*s_i_*))/2 = 0, which simplifies for the symmetric Nash equilibrium with *s_i _*= *s_j _*to

(12) $$v^{\prime }\left(s\right)-\left(f-c+v\left(s\right)\right)=0.$$

In the equilibrium without discrimination, social welfare is

$$W^{o}\left(f\right)=v\left(s\left(f\right)\right)-s\left(f\right)-\theta f$$

since all patients get the same quality *s*(*f*) determined by (12) and the total number of patients is one. The optimal fee *f^o ^*under this regime hence is defined as argmax *_f _W^o^*(*f*) and solves

(13) $$\frac{\partial W^{o}}{\partial f}=\left(v^{\prime }\left(s\left(f^{o}\right)\right)-1\right)\frac{\text{d}s}{\text{d}f}-\theta =0.$$

Differentiating (12) yields d*s*/d*f *= 1/(*v*″(*s*) - *v*'(*s*)) *<*0. The optimality condition (13) hence can only be satisfied for θ *>*0 if *v*'(*s*(*f^o^*)) *<*1. This implies *s*(*f^o^*) *>*0 because *v*'(0) = 1 and *v*″ *<*0. Hence, if balance-billing is not allowed and Medicare pays the optimal fee *f^o ^*all patients receive a service of suboptimal quality compared to the first-best. Glazer and McGuire are able to show that welfare with no discrimination and the optimal fee *f^o ^*is always lower than welfare resulting from the equilibrium with price and quality discrimination and the optimal fee *f^p ^*if θ *>*0.(endnote e)

## 4 The optimal fee under balance billing

Glazer and McGuire are not explicit about how low the second-best optimal fee *f^p ^*that maximizes the welfare function (11) is if balance-billing is allowed. Notably, they do not raise the question whether Medicare should pay any positive fee and, if so, whether the fee should be such that there are any fee-only patients. In the following, we therefore investigate how social surplus changes if Medicare reduces the fee or completely withdraws from the physician market.

In the preceding section, the number of fee-only patients of physician *i *has been shown to be ${\hat{t}}_{i}=\left(p_{i}-s_{i}\right)∕2$ (equation (3)). In the symmetric Nash equilibrium, we obtain from the first-order condition (7) that *p *= (1 + *s *+ *v*(*s*))/2, thus substitution yields ${\hat{t}}_{i}=\left(1-s+v\left(s\right)\right)∕4$. Consequently, the total number of fee-only patients in the market is

(14) $$\hat{t}=\frac{1+v\left(s\right)-s}{2}$$

where *v*(*s*) - *s *≤ 0 is the net social gain from quality, which is decreasing in *s *for *s >*0 because of the concavity of *v*. It is useful to define the level of quality $\bar{s}>0$ at which the number of fee-only patients in (14) becomes zero:

(15) $$1+v\left(\bar{s}\right)-\bar{s}=0$$

Hence, $\bar{s}$ is the upper bound of *s *(a lower bound of quality) such that there are still some fee-only patients in equilibrium. Equation (10) implies that Medicare can always ensure that *s *increases to $\bar{s}$ and hence the number of fee-only patients vanishes by sufficiently decreasing the fee down to $\underline{f}$. The level of $\underline{f}$ can be characterized using the equilibrium condition (9):

(16) $$\underline{f}-c=v^{\prime }\left(\bar{s}\right)\frac{1+v\left(\bar{s}\right)-\bar{s}}{2}-v\left(\bar{s}\right)\Rightarrow \underline{f}=c-v\left(\bar{s}\right)\geq 0,$$

where the definition of $\bar{s}$ in (15) has been used. $\underline{f}$ is thus just as high as the service costs per patient if quality is reduced to its lower bound.

When characterizing the optimal fee under balance billing, Glazer and McGuire restrict themselves to the range between $\underline{f}$ and f* which they define as the 'normal' range of fee policy.(endnote f) It involves a co-existence of price-paying and fee-only patients where the latter receive a service of suboptimal quality. In the following, we first stick to this convention and show in Proposition 1 that $\underline{f}$ is always the optimal fee in this range. Subsequently, we allow a fee policy on the interval [0,∞), i.e., Medicare may completely withdraw from the physician market, and characterize the optimal fee under this regime (Proposition 2).

What is the social surplus if the fee is reduced to $\underline{f}$? Because of $\hat{t}=p-s=0$, the symmetric equilibrium involves a price $p=\bar{s}$ and all patients become price-payers if $Ū-\bar{s}-1∕2\geq 0$ is satisfied, i.e., if the willingness to pay  is sufficiently high.(endnote g) Clearly, all patients then get the socially optimal quality. In order to examine whether this is a welfare optimum, consider the welfare function for the case of price and quality discrimination. It only depends on the quality provided to the fee-only patients. Using (14), it can be written as follows:

(17) $$W^{p}\left(f\right)=\left(v\left(s\left(f\right)\right)-s\left(f\right)\right)\hat{t}\left(s\left(f\right)\right)-\theta f=\left(v\left(s\left(f\right)\right)-s\left(f\right)\right)\frac{1+v\left(s\left(f\right)\right)-s\left(f\right)}{2}-\theta f,$$

where *s *depends on *f *through equation (9). Social surplus is calculated by multiplying per capita net social value of quality with the number of fee-only patients in equilibrium and subtracting the distortion costs of the fee.

**Proposition 1: **If the payer is confined to the normal range of fee policy, i.e. $f\in \left[\underline{f};f^{*}\right]$, and patients' willingness to pay is sufficiently high, then the global welfare maximum is implemented by setting $f=\underline{f}$ for all values of θ *>*0.

**Proof: **We have

$$\frac{\text{d}W^{p}}{\text{d}f}=\left(v^{\prime }\left(s\right)-1\right)\frac{\text{d}s}{\text{d}f}\left(\frac{1}{2}+v\left(s\right)-s\right)-\theta .$$

Evidently, if θ is sufficiently high, then this difference is always negative, and welfare is maximized by setting the lowest possible fee, which is $\underline{f}$. Since this makes the number of fee-only patients in (17) vanish, it induces a welfare level of $-\theta \underline{f}$. Otherwise, there exists a local maximum such that

(18) $$\underset{\geq 0}{\underset{︸}{\left(v^{\prime }\left(s\right)-1\right)\frac{\text{d}s}{\text{d}f}}}\left(\frac{1}{2}+v\left(s\right)-s\right)=\theta .$$

Being confined to *f *≤ f*, we always deal with *s *≥ 0. Hence, by (10), the first two factors at the left-hand side of (18) are both negative. For (18) to be satisfied, 1/2 + *v*(*s*) - *s >*0 is therefore required, which implies $s<\bar{s}$ by (15) and thus $f>\underline{f}$. If it exists, the local maximum is therefore in the interval $\left[\underline{f};f^{*}\right]$ indeed.(endnote h) But as the first term of the welfare function (17), $\left(v\left(s\right)-s\right)\hat{t}\left(s\right)$, can be at most zero due to *v*(*s*) - *s *≤0 and $\hat{t}\geq 0$, any fee higher than $\underline{f}$ must be associated with a welfare level lower than $-\theta \underline{f}$. Hence, even if there exists a local maximum of *W^p^*(*f*) for $f>\underline{f}$, the global welfare maximum in the interval $\left[\underline{f};f^{*}\right]$ is reached at the fee $\underline{f}$. □

The intuition of Proposition 1 can be explained by considering the first-best welfare function $W^{*}\left(f\right)=\left(v\left(s\left(f\right)\right)-s\left(f\right)\right)\hat{t}\left(s\left(f\right)\right)$. It reaches its maximum W* = 0 for two values of *f*. As shown in the preceding subsection, by choosing the first-best fee level f*, quality becomes *s*(f*) = 0 and hence *v*(*s*(f*)) - *s*(f*) = 0. Now, a second possibility to raise the quality component of the welfare function up to zero has been established. This can be achieved by reducing the fee to $\underline{f}$ implying $\hat{t}=0$. It is irrelevant that quality for fee-only patients is minimal under this policy as no patient is affected by it and we are left only with the distortion costs $\theta \underline{f}$ regarding efficiency. Any higher fee with $\hat{t}>0$ and *s *≤ 0 results in a lower social surplus. In terms of the welfare function (11), Medicare should never pay a fee higher than $\underline{f}$.

Obviously, welfare may be further increased by reducing the fee from $\underline{f}$ to zero as this eliminates the distortion costs. However, such a comparison is only valid if all patients continue to be served. We are going to derive the conditions that ensure this in the proof of the following proposition.

**Proposition 2: ***If the payer can set any fee f *∈ [0;∞) and patients' willingness to pay is sufficiently high, then the first-best welfare optimum W* = *W^p ^*= 0 *can be implemented by setting f *= 0 *under balance billing*.

**Proof: **For $f<\underline{f}$, there are no fee-only patients. The market demand faced by physician *i *is consequently determined by the indifferent price-payer who satisfies $Ū-t_{i}^{*}-p_{i}=Ū-\left(1-t_{i}^{*}\right)-p_{j}$. The number of price-paying patients served by *i *is therefore $t_{i}^{*}=\left(1-p_{i}+p_{j}\right)∕2$ and her profit π*^i ^*= (*p_i _*+ *f *- *c*)(1 - *p_i _*+ *p_j_*)/2 with the first-order condition for a Nash equilibrium 1 - *p_j _*- 2*p_i _*+ *c *- *f *= 0. Using symmetry, we can solve for the equilibrium price

(19) p=1+c-f.

If Ū-(1+c)-1∕2≥0, all patients are served if *f *is set to zero.(endnote i) Under this condition, the global welfare optimum W* = *W^p ^*= 0 is therefore attained by setting *f *= 0.□

This establishes that even in the second-best world with θ *>*0, first-best efficiency is implementable by the complete withdrawal of Medicare from the physician market. Provided that the willingness to pay is sufficiently high, all patients then become price-payers and receive the optimal quality while there are no distortion costs so that the surplus function used by Glazer and McGuire becomes maximal. A further implication of Proposition 2 is that 'payer fee discrimination', an alternative fee policy proposed by Glazer and McGuire which discriminates the Medicare fee between fee-only and price-paying patients, cannot further improve efficiency.(endnote j)

**Figure 2:**
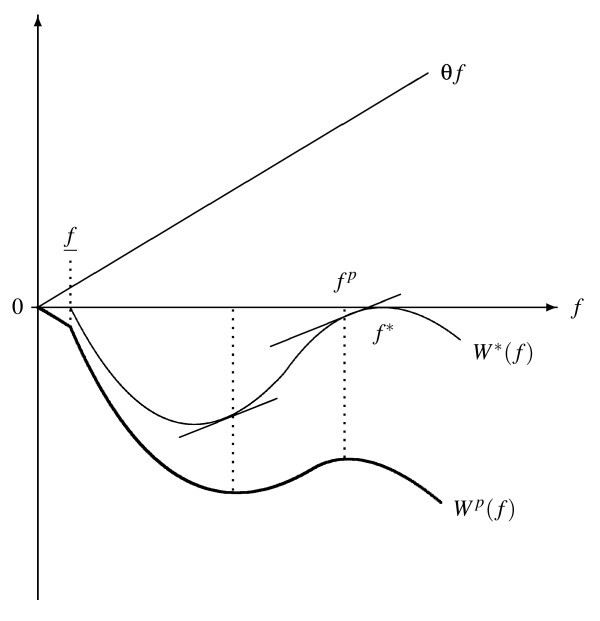
**Second-Best Welfare and Medicare payments *f***.

Figure 2 illustrates the shape of the welfare function (17). The graph of *W^p ^*(*f*) is constructed as the difference between the first-best welfare W*(*f*) = (*v*(*s*(*f*)) - *s*(*f*))(1 + *v*(*s*) - *s*(*f*))/2 and the distortion costs θ*f*. W* has a corner maximum on the interval $f\in \left[0;\underline{f}\right]$ with W*(*f*) = 0 as well as an interior maximum for *f *= f* with W*(f*) = 0.(endnote k) Subtracting θ*f*, we are left with a corner maximum for *f *= 0 with *W^p^*(0) = 0 and a local maximum for *f^p ^*to the left of f* with *W^p^*(*f^p^*) *<*0.(endnote l) The welfare considerations of Glazer and McGuire are based on the existence of both fee-only patients and price-payers, as it is associated with the fee *f^p ^>*0. However, this is only a local, not the global second-best optimum.

The central result obtained by Glazer and McGuire must therefore be strengthened: moving from an equilibrium without balance-billing and optimal fee *f^o ^*to an equilibrium with balance-billing and optimal fee *f *= 0, not only a welfare improvement but the first-best optimum can be attained. As we show in the following section, however, this result depends crucially on the welfare function (11) which only takes into account the efficiency of quality but does not consider any distributional effects. From the patients' perspective, a Medicare fee in the 'normal range' $\left[\underline{f};f^{*}\right]$ can be optimal.

## 5 Balance billing and patient welfare

An important aspect with respect to balance billing is how patients are affected by this policy. In particular, there is the concern that patients are made worse off if physicians are allowed to charge an additional price. In the models by Paringer, Mitchell and Cromwell as well as Zuckerman and Holahan which do not consider effects on quality, this can lead to the drastic effect that balance billing only raises the physician's rent [2-4]. An open question is the effect on patient welfare within the model by Glazer and McGuire. Is the positive effect of balance billing on quality dominant or are the quality gains transformed into higher rents for physicians? In this section, we take the patients' point of view and try to answer this question. Before we ask in Section 5.2 whether balance billing should be allowed, we first determine in Section 5.1 the optimal Medicare fee if balance billing is allowed. Finally, we consider the effects of 'fee discrimination', a policy proposed by Glazer and McGuire, on patient welfare in Section 5.3.

### 5.1 Should Medicare set a positive fee under balance billing?

To assess the effects of Medicare's policy on patient welfare, we need to specify in more detail how Medicare's expenditures are financed. In the following, we assume that the government collects a uniform contribution (1 + θ)*f *from each individual where *f *is the fee paid to physicians and θ*f *are the administrative costs of Medicare per capita. Hence, the utility for fee-only patients is given by U=Ū-t-s-(1+θ)f while price-paying patients obtain utility $U^{p}=Ū-t-p-\left(1+\theta \right)f$.

If Medicare sets a fee *f *above $\underline{f}$, then

(20) $$\tilde{t}=\frac{1-p+s}{2}<\frac{1}{2}\;\Leftrightarrow \;p>s.$$

**Figure 3:**
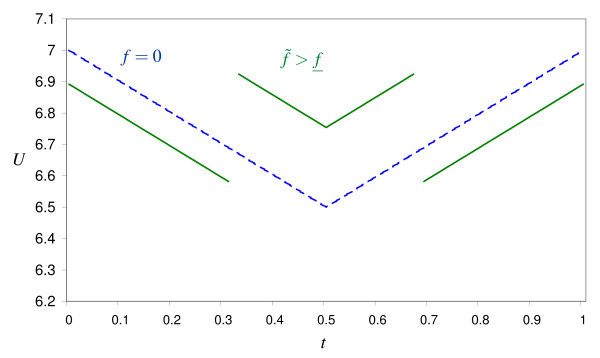
**Utility distribution for *f *= 0 and $\tilde{f}>\underline{f}$(Ū=10, *c *= 2, *a *= 1, θ = 20%, $\tilde{f}=1.59$, $\underline{f}=1.16$)**.

Thus, patients who are treated at the fee only face a lower quality reduction than the price charged from price-paying patients. This is illustrated in Figure 3 which is based on the cost savings function

(21) $$v\left(s\right)=-\exp\left(-as-\ln\left(a\right)\right)+a^{-1}$$

where we set *a *= 1. It shows the utility distribution for *f *= 0 and for a fee $\tilde{f}>\underline{f}$. Price-paying patients are worse off and fee-only patients are better off for $\tilde{f}>\underline{f}$ compared to *f *= 0.

We measure patient welfare by the sum of utility of all patients.(endnote m) As we show in Appendix A.1, patient welfare under balance billing *PW^p ^*then corresponds to

(22) $$PW^{p}\left(f\right)=\left\{\begin{array}{cc} Ū-\frac{1}{4}-\left(1+c\right)-\theta f & \text{if}\;0\leq f<\underline{f}\\ Ū-\frac{1}{4}-p\left(f\right)+{\left(s\left(f\right)-p\left(f\right)\right)}^{2}-\left(1+\theta \right)f & \text{if}\;\;\;\;f\geq \underline{f}. \end{array}\right)$$

Increasing Medicare's fee therefore lowers patient welfare if $f\in \left[0;\underline{f}\right)$ provided that θ *>*0. In this range, all patients are balance billed and raising *f *reduces the price *p *to the same extent since *p *= 1 + *c *- *f *by equation (19). The net effect is therefore a fall in utility by higher administrative costs. To see whether a positive fee can increase patient welfare we can therefore limit ourselves to the case in which $f\geq \underset{-}{f}$ and analyze the following difference

$$PW^{p}\left(f\geq \underline{f}\right)-PW^{p}\left(f=0\right)=h\left(f,\theta \right)+{\left(s\left(f\right)-p\left(f\right)\right)}^{2}$$

where

(23) h(f,θ)≡1+c-p(f)-(1+θ)f.

If $PW^{p}\left(f\geq \underline{f}\right)-PW^{p}\left(f=0\right)>0$, a positive Medicare fee raises patient welfare. Note that a positive value of the function *h*(*f*, θ) is sufficient for this result since (*s*(*f*) - *p*(*f*))^2 ^is nonnegative.

By equations (7) and (19) we obtain for the price for balance-billed patients

$$p\left(f\right)=\left\{\begin{array}{cc} 1+c-f & \text{if }0\leq \;f<\underline{f}\\ \frac{1+s\left(f\right)+v\left(s\left(f\right)\right)}{2} & \text{if}\;\;\;\;f\geq \underline{f}. \end{array}\right)$$

This yields *h*(*f*, θ) = 0 for θ = 0 and $f<\underline{f}$. For θ = 0 and $f\geq \underset{-}{f}$, we obtain

$$h\left(f,\theta \right)=1+c-\frac{1+s\left(f\right)+v\left(s\left(f\right)\right)}{2}-f.$$

In Appendix A.2 we show that *h*(*f*) is increasing in *f *at $f=\underline{f}$ if θ = 0. Thus, without administrative costs, increasing *f *beyond $\underline{f}$ implies 1 + *c > p*(*f*) + (1 + θ) *f *and patient welfare increases. Noting that

$$\frac{\partial h\left(f,\theta \right)}{\partial \theta }=-f<0,$$

this result continues to hold as long as θ is below a critical value ${\hat{\theta }}_{PW}$. Furthermore, a positive value of *h*(*f*, θ) for $f>\underline{f}$ is equivalent to

p(f)+(1+θ)f<1+c=p(f=0).

**Figure 4:**
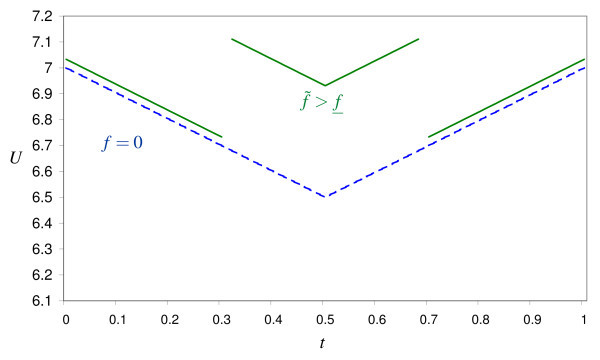
**Utility distribution for *f *= 0 and $f>\underline{f}$(Ū=10, *c *= 2, *a *= 1, θ = 10%, $\tilde{f}=1.67$, $\underline{f}=1.16$)**.

Thus, for small values of θ even price-paying patients are better off if *f *is raised above $\underline{f}$. Since by (20) fee-only patients face a lower quality reduction than the price charged from price-paying patients, all patients must therefore be better off. This is shown in Figure 4 which relies on the same parameters as Figure 3 except θ which is 10% instead of 20%.(endnote n)

We summarize our results in

**Proposition 3: ***Under balance-billing setting*, $f>\underline{f}$*increases patient welfare if Medicare's administrative cost markup *θ *is smaller than a critical value *${\hat{\theta }}_{PW}>0$. *Even all patients can be better off if *θ *is sufficiently small*.

Table 1 shows the results for a numerical simulation with the cost savings function (21) for *a *= 1. Patient welfare if the Medicare fee is zero is given by *PW^p^*(*f *= 0). For different values of θ, the Medicare fee ${\tilde{f}}^{*}$ which maximizes patient welfare is calculated conditional on ${\tilde{f}}^{*}>\underline{f}$. $PW^{p}\left({\tilde{f}}^{*}\right)$ is the corresponding patient welfare. The simulation shows that

**Table 1:** Patient welfare under balance billing Ū=10, *c *= 2, *a *= 1.

θ	${\tilde{f}}^{*}$ for $f>\underline{f}$	$p\left({\tilde{f}}^{*}\right)+\left(1+\theta \right){\tilde{f}}^{*}$	1 + *c*	$PW^{p}\left({\tilde{f}}^{*}\right)$	*PW ^p^*(*f *= 0)
0%	1.86	2.82	3	7.13	6.75
10%	1.75	2.98	3	6.95	6.75
20%	1.66	3.13	3	6.78	6.75
30%	1.59	3.27	3	6.61	6.75
40%	1.52	3.40	3	6.46	6.75
50%	1.47	3.54	3	6.31	6.75

• for θ = 0% or 10%, we have $1+c>p\left({\tilde{f}}^{*}\right)+\left(1+\theta \right){\tilde{f}}^{*}$. Thus, all patients are better off by setting the fee above $\underline{f}$.

• for θ = 0% to 20%, we have $PW^{p}\left({\tilde{f}}^{*}\right)>PW^{p}\left(f=0\right)$, i.e., patient welfare is higher for a fee above $\underline{f}$. The critical value is ${\hat{\theta }}_{PW}=21.5\%$.

**Figure 5:**
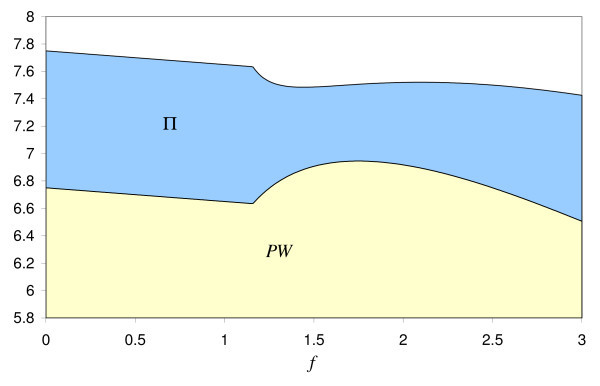
**Patient welfare and profits as a function of *f*, θ = 10%**.

Our result is in stark contrast to the social surplus analysis in section 4 where *f *= 0 is the optimal fee level. In particular, setting the Medicare fee above $\underline{f}$ implies that some patients receive suboptimal quality. However, the decrease of average quality is not the only effect of an increase in Medicare's fee. Furthermore, profits of physicians are affected. Denoting aggregate profits by Π, we show in Appendix A.3 that the following relation holds

(24) $$-{\left(\frac{\text{d}\Pi }{\text{d}f}\right|}_{f=\underline{f}}>{\left(\frac{\text{d}PW^{p}\left(\theta =0\right)}{\text{d}f}\right|}_{f=\underline{f}}>0.$$

i.e., the decrease in physicians's profits is larger than the increase in patient welfare. For our numerical simulation with θ = 10%, Figure 5 shows how patient welfare increases even though social surplus falls. Patients are better off even though quality provision is less efficient on average.

This result can be explained by the effects of Medicare's policy on physician competition. By raising *f*, competition for patients gets more intense and profits of physicians fall by more than the decrease in average quality. Especially patients in the middle get a better deal as physicians are willing to treat them at the fee-only. Although they receive lower quality, they get a more favorable offer than price-paying patients as *p > s*. In addition, price-paying patients may also be better off if Medicare's administrative cost markup θ is sufficiently small.

### 5.2 Should balance billing be allowed?

One of the central findings of Glazer and McGuire is that social surplus is generally higher if balance billing is allowed. However, balance billing also gives physicians the opportunity to increase their profits. It is therefore unclear whether patients also benefit if balance billing is permitted. We investigate this issue by taking a regime without balance billing and *f^o^*, the corresponding optimal fee from an efficiency perspective, as a reference point (see equation (13)). The corresponding quality level *s^o^*(*f^o^*) is defined by equation (12), leading to patient welfare

(25) $$PW^{o}\left(f^{o}\right)=Ū-\frac{1}{4}-s^{o}\left(f^{o}\right)-\left(1+\theta \right)f^{o}.$$

Turning to a comparable regime with balance billing, we define ${\hat{f}}^{p}$ as the solution to $s^{p}\left({\hat{f}}^{p}\right)=s^{o}$, i.e., the value of *f^p ^*which leads to the same quality under balance billing. From equation (9) which holds in the symmetric equilibrium with balance billing, we can infer that

(26) $${\hat{f}}^{p}=v^{\prime }\left(s^{o}\right)\frac{1+v\left(s^{o}\right)-s^{o}}{2}-v\left(s^{o}\right)+c.$$

First, we assume that ${\hat{f}}^{p}>\underline{f}$. If balance billing is prohibited, then equation (12) holds in equilibrium which implies *f^o ^*= -*v*(*s^o^*) + *c *+ *v*'(*s^o^*). Inserting in (26) leads to

$$f^{o}-{\hat{f}}^{p}=v^{\prime }\left(s^{o}\right)\frac{1+s^{o}-v\left(s^{o}\right)}{2}>0$$

since *s^o ^*- *v*(*s^o^*) ≥ 0 is implied by *v*(0) = 0,*v*'(0) = 1, *v*'(*s*) *>*0 and *v*″(*s*) *<*0. Thus, under balance billing the same quality for fee-only patients can be provided with a lower Medicare fee.

For patient welfare under balance billing (see equation (22)), we obtain

$$PW^{p}\left({\hat{f}}^{p}>\underline{f}\right)=Ū-\frac{1}{4}-p\left({\hat{f}}^{p}\right)+{\left(s^{o}-p\left({\hat{f}}^{p}\right)\right)}^{2}-\left(1+\theta \right){\hat{f}}^{p}.$$

Using (25) yields for the difference of total utilities under the two regimes

(27) $$PW^{p}\left({\hat{f}}^{p}>\underline{f}\right)-PW^{o}\left(f^{o}\right)=\left(s^{o}-p\left({\hat{f}}^{p}\right)\right)+{\left(s^{o}-p\left({\hat{f}}^{p}\right)\right)}^{2}+\left(1+\theta \right)\left(f^{o}-{\hat{f}}^{p}\right).$$

The first term is negative since *s > p *for ${\hat{f}}^{p}>\underline{f}$ (see equation (20)), the second term is nonnegative, the third term strictly positive. Thus, this difference is positive for any value of *s^o ^*if θ is sufficiently large. Allowing balance billing is then superior from the patients' perspective. It can always replicate the level of quality at a lower cost which is sufficient to increase patient welfare.

Next, we turn to the case ${\hat{f}}^{p}\leq \underline{f}$. In this case, the best choice under balance billing is to set *f^p ^*= 0 as there are only price-paying patients. This yields *p *= 1 + *c *and

$$PW^{p}\left(f^{p}=0\right)-PW^{o}\left(f^{o}\right)=-\left(1+c\right)-s^{o}\left(f^{o}\right)-\left(1+\theta \right)f^{o}.$$

Again if θ is sufficiently large, then average utility increases when balance billing is allowed.(endnote o) We can therefore conclude in

**Proposition 4: ***For a given fee level f^o ^without balance billing, patient welfare can be increased by allowing balance billing if Medicare's administrative cost markup *θ *is sufficiently large*.

Proposition 4 shows the main drawback of prohibiting balance billing. Inducing quality without balance billing is very costly if Medicare's administrative costs are high. Permitting balance billing allows to induce the same quality at a lower Medicare fee. The corresponding savings in administrative costs can exceed higher payments to physicians from patients who are balance billed.

In assessing Proposition 4, however, one has to keep in mind that neither *f^o ^*nor ${\hat{f}}^{p}$ are chosen optimally, i.e., maximize patient welfare for each regime. With respect to the regime without balance billing, this opens the possibility that *f^o ^*is not the optimal choice for values of θ which allow higher patient welfare under balance billing. On the other hand, under balance billing patient welfare is generally higher by setting a fee different from ${\hat{f}}^{p}$.

Numerical simulations based the cost savings function (21) indicate that Proposition 4 can also be extended to optimally chosen fee levels. An example is shown in Table 2. For different values of θ, *s*^o^* and *f*^o^* are the optimal values without balance billing. Maximized patient welfare without balance billing is denoted by *PW^o^*(*f*^o^*). Besides ${\hat{f}}^{p}$ also the fee level *f*^p* ^which maximizes patient welfare with balance billing is given as well as the corresponding total utilities $PW^{p}\left({\hat{f}}^{p}\right)$ and *PW^p^*(*f*^p^*). If θ *>*16.7%, then $PW^{p}\left({\hat{f}}^{p}\right)>PW^{o}\left(f^{o*}\right)$ and balance billing with the same quality for fee-only patients is superior to an optimal regime without balance billing. For θ *>*1.2%, *PW^p^*(*f *^p^*) *> PW^o^*(*f*^o^*), i.e., prohibiting balance billing yields higher patient welfare only for low values of administrative costs. Note that for θ = 10% and 20%, balance billing with a positive Medicare fee in the 'normal range' $\left[\underline{f};f^{*}\right]$ is optimal. For θ = 30% and 40%, we have *f*^p* ^= 0. In this case, all patients are better off if balance billing is permitted.

**Table 2:** Prohibiting vs.allowing balance billing Ū=10, *c *= 2, *a *= 1.75, $\underline{f}=1.47$, f * = 1.91.

θ	*s*^o^*	*f*^o^*	${\hat{f}}^{p}$	*f*^p^*	*PW^o^*(*f*^o^*)	$PW^{p}\left({\hat{f}}^{p}\right)$	*PW ^p^*(*f*^p^*)
0%	0.58	2.00	1.78	1.91	7.172	7.154	7.171
10%	0.63	1.95	1.74	1.85	6.975	6.967	6.983
20%	0.69	1.90	1.71	1.81	6.782	6.786	6.800
30%	0.73	1.87	1.68	0	6.593	6.607	6.750
40%	0.77	1.84	1.66	0	6.408	6.433	6.750

**Figure 6:**
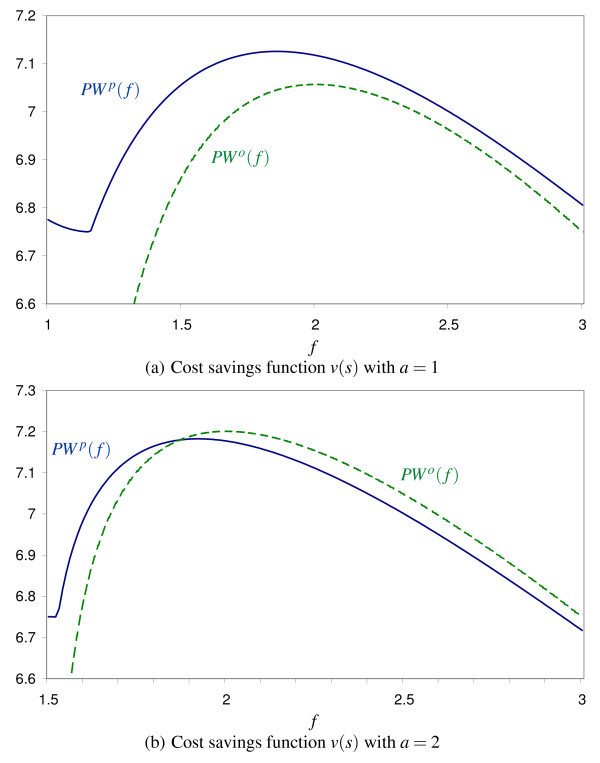
**Patient welfare as a function of *f*, θ = 0**.

It is also possible that allowing balance billing yields higher patient welfare already for **θ **= 0. The shape of the cost savings function *v*(*s*) is crucial for this result. This is shown in Figure 6. Setting *a *= 2 in the cost savings function (21) yields the optimal values *f*^o^* = 2.00, *f*^p* ^= 1.92, *PW^o^*(*f*^o^*) = 7.201 and *PW^p^*(*f*^p^*) = 7.183 which implies that prohibiting balance billing is superior from the patients' perspective (see Figure 6(b)). For *a *= 1, however, we obtain *f *^o^* = 2.00, *f*^p* ^= 1.86, *PW^o^*(*f*^o^*) = 7.057 and *PW^p^*(*f*^p^*) = 7.126. Figure 6(a) shows that allowing balance billing is superior for all values of *f *if θ = 0.

In sum, it depends on Medicare's administrative costs and the properties of the cost function whether allowing balance billing raises patient welfare. In contrast to Glazer and McGuire, we do not find that allowing balance billing is generally superior. In their analysis, only quality effects matter and allowing balance billing is better because it can always induce the same quality at a lower cost. From the patients' perspective, it also has to be taken into account that physicians charge a price from selected patients. This reduces patient welfare. As long as θ is small and inducing quality without balance billing is therefore not too costly, this profit effect may dominate and prohibiting balance billing leads to higher patient welfare. For example, if *a *= 2 and θ = 0, aggregate profits are Π*^o^*(*f*^o^*) = 0.333 and Π*^p^*(*f *^p^*) = 0.513. But higher profits do not necessarily imply lower patient welfare. For *a *= 1 and θ = 0, the corresponding values are Π*^o^*(*f*^o^*) = 0.5 and Π*^p^*(*f*^p^*) = 0.574. Nevertheless, patient welfare is higher as the Medicare fee is lower (1.86 vs. 2.00) and the quality reduction for fee-only patients is significantly smaller (0.52 vs. 0.69) under balance billing.

### 5.3 Fee discrimination

Glazer and McGuire also analyze a regime of balance billing under which Medicare discriminates the fee depending on whether the physician treats patients at the fee only or balance bills them. Under this policy, physicians are reimbursed *f *+ *d *if they do not charge their patients price *p *and *f *- *d *if they do, with *d >*0. Glazer and McGuire argue that such a fee policy is welfare improving based on the efficiency criterion if the extent of discrimination as measured by *d *is small and if the fee is set close to its optimum.(endnote p) However, as already mentioned in Section 4, Proposition 2 implies that the first-best welfare level can be implemented by Medicare withdrawing form the market, ruling out any strictly positive efficiency effect of fee discrimination. The question remains to be answered whether fee discrimination can be justified if Medicare is assumed to be concerned about patient welfare.

We first derive the equilibrium price under fee discrimination. Since the payer increases the fee by *d >*0 for the treatment of fee-only patients and reduces it by the same amount in the other cases, physician *i*'s profit becomes

$$\pi^{i}=\left(f-d+p_{i}-c\right)\frac{1-p_{i}+s_{j}}{2}+\left(f+d-c+v\left(s_{i}\right)\right)\frac{p_{i}-s_{i}}{2}$$

with the first-order conditions for a Nash-equilibrium

(28) $$\frac{1-p_{i}+s_{j}}{2}-\frac{f-d+p_{i}-c}{2}+\frac{f+d-c+v\left(s_{i}\right)}{2}=0$$

(29) $$-\frac{f+d-c+v\left(s_{i}\right)}{2}+v^{\prime }\left(s_{i}\right)\frac{p_{i}-s_{i}}{2}=0.$$

Assuming symmetry, (28) can be solved for the equilibrium price under fee discrimination

(30) $$p\left(d\right)=\frac{1+s\left(d\right)+v\left(s\left(d\right)\right)}{2}+d.$$

Thus, two effects result from the introduction of fee discrimination by *d *on the price physicians charge. First, a direct effect implies that physicians increase the price just by *d *to compensate for the lower fee that they receive for the treatment of price-paying patients. Note that this is a difference to variations in *f *where no such direct effect on the price exists. Second, an indirect effect on *p *works through the influence of *d *on the equilibrium level of quality. By substituting (30) into (29) for the symmetric case and differentiating, we can determine this effect of *d *on quality as follows

(31) $$\frac{\text{d}s}{\text{d}d}=\frac{2}{v^{\prime \prime }\left(s\right)\left(1-s+2d+v\left(s\right)\right)-3v^{\prime }\left(s\right)+{\left(v^{\prime }\left(s\right)\right)}^{2}}\left(1-v^{\prime }\left(s\right)\right).$$

Hence, (31) is similar to d*s*/d*f *in (10) with one difference. In contrast to (10), the sign of (31) now depends on the volume of the marginal cost savings from reduced quality *v*'(*s*). This results from the direct effect of *d *on the price found in (30). If marginal cost savings are high (*v*'(*s*) *>*1 ⇔ *s <*0), then quality is decreased in response to a marginal increase in *d *and the price is increased more than proportionally. This can be seen from differentiating (30)

$$\frac{\text{d}p}{\text{d}d}=\frac{1+v^{\prime }\left(s\right)}{2}\frac{\text{d}s}{\text{d}d}+1.$$

Otherwise, due to (10) and (31), physicians react to an increase in the amount of fee discrimination by an enhanced quality and a less than compensating price increase. In this case, a marginal increase in *d *is similar to an increase of the fee *f *which induces physicians to offer a better quality and decrease the price.

Given this information, we are able to compute the total number of fee-only patients

$$2\hat{t}=p-s\left(d\right)=\frac{1-s\left(d\right)+2d+v\left(s\left(d\right)\right)}{2}$$

and of price-paying patients in the market with fee discrimination

(32) $$2\tilde{t}=\frac{1+s\left(d\right)-2d-v\left(s\left(d\right)\right)}{2}.$$

In the absence of fee discrimination (*d *= 0), we have $2\tilde{t}\geq 1∕2$ due to *s *- *v*(*s*) ≥ 0 meaning that we always observe more price payers than fee-only patients in the market except for *s *= 0 where their numbers are just equal. Introducing fee discrimination, however, decreases the number of price paying patients. Indeed, differentiating (32) yields

$$\frac{\text{d}\tilde{t}}{\text{d}d}=\frac{1}{2}\left(\frac{1-v^{\prime }\left(s\right)}{2}\frac{\text{d}s}{\text{d}d}-1\right),$$

which is unambiguously negative. Intuitively, fee discrimination with *d >*0 makes fee-only patients more attractive to physicians. Therefore, their number rises in equilibrium.

These expressions allow us to compare patient welfare with fee discrimination (*FD*) and without fee discrimination (*NFD*) under balance billing. Assuming $f\geq \underset{-}{f}$ we show in Appendix A.4 that the change in patient welfare induced by fee discrimination is

(33) $$\begin{array}{ll} \Delta PW & =PW^{FD}-PW^{NFD}\;\\ & =\frac{1+s-v\left(s\right)}{2}\frac{1-s+v\left(s\right)}{2}\;\\ & \;-\frac{1+s\left(d\right)-2d-v\left(s\left(d\right)\right)}{2}\times \frac{1-s\left(d\right)+2d+v\left(s\left(d\right)\right)}{2}\;\\ & \;+s-s\left(d\right)-\left(1+\theta \right)d\left(2d+v\left(s\left(d\right)\right)-s\left(d\right)\right).\;\\ & \; \end{array}$$

Clearly, this is zero for *d *= 0 and differentiation yields after some rearrangements

(34) $$\frac{\text{d}\Delta PW}{\text{d}d}{|}_{d=0}=-\frac{\text{d}s}{\text{d}d}{|}_{d=0}+\frac{1-v^{\prime }\left(s\right)}{2}\frac{\text{d}s}{\text{d}d}{|}_{d=0}\left(s-v\left(s\right)\right)+\theta \left(s-v\left(s\right)\right),$$

Based on this equation, we prove the following in Appendix A.5.

**Proposition 5: ***A small amount of payer fee discrimination increases patient welfare if either*

• θ *is sufficiently high, or*

• *the fee is chosen so as to maximize patient welfare and this results in s >*0.

Under these circumstances, Medicare paying a higher fee to physicians who renounce on balance billing can indeed be justified from the perspective of the patients. However, it cannot be excluded that fee discrimination lowers patient welfare. This may occur whenever the price increase for the price payers dominates the quality increase for the fee-only patients or if the fee is set so that *s <*0, which implies that fee discrimination actually lowers quality.

## 6 Conclusions

This paper has revisited the economics of 'balance billing' in the framework by Glazer and McGuire [1]. We analyzed the optimal Medicare policy from the perspective of patients and showed that a positive Medicare fee and a mixed system with price-paying and fee-only patients can increase patient welfare under balance billing if the administrative costs of Medicare are sufficiently low. The intuition for this result is that a positive Medicare fee increases competition of physicians which lowers the total payment to physicians by Medicare and patients.

Furthermore, we examined the case for permitting balance billing. We showed that it depends on Medicare's administrative costs and the properties of the physicians' cost function whether allowing balance billing raises patient welfare. In contrast to Glazer and McGuire, we do not find that allowing balance billing is generally superior as balance billing allows physicians to increase their rents. However, both physicians' rents and patient welfare can be higher if balance billing is permitted. This is the case for sufficiently high administrative costs of Medicare. For some cost functions, patient welfare can be higher under balance billing even in the absence of administrative costs.

Finally, we considered the effects on patient welfare if Medicare discriminates the fee depending on whether the physician treats patients at the fee only or balance bills them. This policy can also help to raise patient welfare. This is the case if Medicare's administrative costs are high or if Medicare's optimal fee under balance billing implies lower quality for fee-only patients.

Our study relied on a model based on profit-maximizing physicians. It may be interesting to relax this assumption in future research. Altruistic physicians may be less inclined to provide lower quality to fee-only patients. Furthermore, we assumed symmetric information about the quality of physicians' services. To the extent that patients cannot judge the quality of services, the efficiency of balance billing may be questionable. Balance-billed patients may only receive non-medical amenities such as shorter waiting times for non-urgent treatments. An interesting extension is also to allow patients to differ in ability to pay.

## Competing interests

The authors declare that they have no competing interests.

## Authors' contributions

MK and FS have carried out the analysis and written the paper together. Both authors read and approved the final manuscript.

## Appendix

### A.1 Patient welfare under balance billing

For $f<\underline{f}$, patient welfare is given by

$$\begin{array}{c} PW^{p}\left(f\right)=2\int_{0}^{1∕2}\;Ū-t-p-\left(1+\theta \right)f\text{d}t\\ =Ū-\frac{1}{4}-\left(1+c\right)-\theta f \end{array}$$

as *p *= 1 + *c *- *f *in this case. If $f\geq \underset{-}{f}$, we obtain

$$\begin{array}{ll} PW^{p}\left(f\right) & =2\left\{\int_{0}^{\tilde{t}\left(f\right)}Ū-t-p\left(f\right)-\left(1+\theta \right)f\text{d}t+\int_{\tilde{t}\left(f\right)}^{1∕2}Ū-t-s\left(f\right)-\left(1+\theta \right)f\text{d}t\right\}\;\\ & =Ū-\frac{1}{4}+2\tilde{t}\left(f\right)\left(s\left(f\right)-p\left(f\right)\right)-s\left(f\right)-\left(1+\theta \right)f.\;\\ & \; \end{array}$$

Using $\tilde{t}\left(f\right)=\left(1-p+s\right)∕2$ according to (1), this simplifies to

$$PW^{p}\left(f\right)=Ū-\frac{1}{4}-p\left(f\right)+{\left(s\left(f\right)-p\left(f\right)\right)}^{2}-\left(1+\theta \right)f.$$

Thus, patient welfare is

(A22) $$PW^{p}\left(f\right)=\left\{\begin{array}{cc} Ū-\frac{1}{4}-\left(1+c\right)-\theta f & \text{if}\;0\leq f<\underline{f}\\ Ū-\frac{1}{4}-p\left(f\right)+{\left(s\left(f\right)-p\left(f\right)\right)}^{2}-\left(1+\theta \right)f & \text{if}\;\;\;\;f\geq \underline{f}. \end{array}\right)$$

### **A.2 Properties of the function ***h*(*f*)

In the following, we show that the function

(A23) h(f,θ)≡1+c-p(f)-(1+θ)f.

is increasing in *f *at $f=\underline{f}$ if θ = 0. We have

$$\frac{\partial h\left(\underline{f},\theta =0\right)}{\partial f}=-\frac{1+v^{\prime }\left(s\left(\underline{f}\right)\right)}{2}{\left(\frac{\text{d}s}{\text{d}f}\right|}_{f=\underline{f}}-1,$$

At $f=\underline{f}\Rightarrow s=\bar{s}$ and therefore $1+v\left(\bar{s}\right)=\bar{s}$. From equation (10), we obtain

$${\left(\frac{\text{d}s}{\text{d}f}\right|}_{f=\underline{f}}=\frac{2}{{\left(v^{\prime }\left(\bar{s}\right)\right)}^{2}-3v^{\prime }\left(\bar{s}\right)}$$

and therefore

$$\frac{\partial h\left(\underline{f},\theta =0\right)}{\partial f}=\frac{1+v^{\prime }\left(\bar{s}\right)}{3v^{\prime }\left(\bar{s}\right)-{\left(v^{\prime }\left(\bar{s}\right)\right)}^{2}}-1.$$

Since $\bar{s}>0$, we have $v^{\prime }\left(\bar{s}\right)<1$. Furthermore, we must have

$$\frac{1+v^{\prime }\left(\bar{s}\right)}{3v^{\prime }\left(\bar{s}\right)-{\left(v^{\prime }\left(\bar{s}\right)\right)}^{2}}>1$$

as the function *g*(*a*) = (1 + *a*)/(3*a *- *a*^2^) has the following properties: *g*(1) = 1 and *g*'(*a*) = (*a*^2 ^+ 2*a *- 3)(3*a *- *a*^2^)^-2 ^*<*0 for 0 ≤ *a <*1. Thus, *g*(*a*) *>*1 for 0 ≤ *a <*1 and

(A.1) $$\frac{\partial h\left(f,\theta =0\right)}{\partial f}=-\frac{1+v^{\prime }\left(s\left(\underline{f}\right)\right)}{2}{\left(\frac{\text{d}s}{\text{d}f}\right|}_{f=\underline{f}}-1>0.$$

If θ = 0, increasing *f *beyond $\underline{f}$ therefore implies 1 + *c > p*(*f*) + (1 + θ) *f *and all patients are better off. Noting that

$$\frac{\partial h\left(f,\theta \right)}{\partial \theta }=-f<0,$$

this result continues to hold as long as θ is below a critical value ${\hat{\theta }}_{ALL}$.

### A.3 Physicians' profits under balance billing

Aggregate profits are given by (cf. (4))

$$\begin{array}{ll} \Pi & =2\left(\left(p+f-c\right)\tilde{t}+\left(f-c+v\left(s\right)\right)\left(0.5-\tilde{t}\right)\right)\;\\ & =2p\tilde{t}+v\left(s\right)\;\left(1-2\tilde{t}\right)+f-c.\;\\ & \; \end{array}$$

For $f<\underline{f}$ and therefore *p *= 1 + *c *- *f *and $\tilde{t}=1∕2$, this simplifies to Π = 1. For $f\geq \underset{-}{f}$, we obtain with $\tilde{t}=\left(1-p+s\right)∕2$:

$$\begin{array}{ll} \Pi & =p\left(1-p+s\right)+v\left(s\right)\;\left(p-s\right)+f-c\;\\ & =\left(p-v\left(s\right)\right)\;\left(1-p+s\right)+v\left(s\right)+f-c.\;\\ & \; \end{array}$$

Inserting *p *= (1 + *s *+ *v*(*s*))/2 from (7) yields

$$\begin{array}{c} \Pi =\frac{1+s+v(s)}{2}\frac{1+s-v(s)}{2}+v(s)\frac{1-s+v(s)}{2}+f-c\\ =\frac{{\left(1+s\right)}^{2}-{\left(v(s)\right)}^{2}}{4}+\frac{2v(s)-2sv(s)+2(v(s{))}^{2}}{4}+f-c\\ =\frac{{\left(1+s\right)}^{2}+{\left(v(s)\right)}^{2}}{4}-\frac{2v(s)(s-1)}{4}+f-c\\ ={\left(\frac{1+s-v(s)}{2}\right)}^{2}+v(s)+f-c. \end{array}$$

Thus, we can summarize

$$\Pi \left(f\right)=\left\{\begin{array}{cc} 1 & \text{if }0\leq \;f<\underline{f}\\ {\left(\frac{1+s-v\left(s\right)}{2}\right)}^{2}+v\left(s\right)+f-c & \text{if}\;\;\;f\geq \underline{f}. \end{array}\right)$$

For $f\geq \underset{-}{f}$ we obtain

$$\begin{array}{ll} \frac{\text{d}\Pi }{\text{d}f} & =\left(1+s-v\left(s\right)\right)\left(1-v^{\prime }\left(s\right)\right)\frac{\text{d}s}{\text{d}f}+v^{\prime }\left(s\right)\frac{\text{d}s}{\text{d}f}+1\;\\ & =\left(1+s-v\left(s\right)-v^{\prime }\left(s\right)\left(s-v\left(s\right)\right)\right)\frac{\text{d}s}{\text{d}f}+1.\;\\ & \; \end{array}$$

At $f=\underline{f}$, we have $\bar{s}-v\left(\bar{s}\right)=1$ and therefore

$${\left(\frac{\text{d}\Pi }{\text{d}f}\right|}_{f=\underline{f}}=\left(2-v^{\prime }\left(\bar{s}\right)\right){\left(\frac{\text{d}s}{\text{d}f}\right|}_{f=\underline{f}}+1.$$

Patient welfare for $f\geq \underset{-}{f}$ is given by equation (22). Using *p *= (1 + *s *+ *v*(*s*))/2 from (7) yields

$$PW^{p}\left(f\right)=Ū-\frac{1}{2}+\frac{{\left(s\left(f\right)-v\left(s\left(f\right)\right)\right)}^{2}}{4}-s\left(f\right)-\left(1+\theta \right)f$$

and therefore

$${\left(\frac{\text{d}PW^{p}}{\text{d}f}\right|}_{f=\underline{f}}=-\frac{1+v^{\prime }\left(\bar{s}\right)}{2}{\left(\frac{\text{d}s}{\text{d}f}\right|}_{f=\underline{f}}-\left(1+\theta \right).$$

Equation (A.1) implies

$${\left(\frac{\text{d}PW^{p}\left(\theta =0\right)}{\text{d}f}\right|}_{f=\underline{f}}=-\frac{1+v^{\prime }\left(\bar{s}\right)}{2}{\left(\frac{\text{d}s}{\text{d}f}\right|}_{f=\underline{f}}-1>0.$$

Furthermore,

$$\frac{1+v^{\prime }\left(\bar{s}\right)}{2}<2-v^{\prime }\left(\bar{s}\right)$$

since $v^{\prime }\left(\bar{s}\right)<1$. Thus,

(A24) $$-{\left(\frac{\text{d}\Pi }{\text{d}f}\right|}_{f=\underline{f}}>{\left(\frac{\text{d}PW^{p}\left(\theta =0\right)}{\text{d}f}\right|}_{f=\underline{f}}>0,$$

### A.4 Patient welfare under fee discrimination

As we have shown in Appendix A.1, patient welfare under balance billing without fee discrimination (*NFD*) is

$$PW^{NFD}\left(f>\underline{f}\right)=Ū-1∕4+2\tilde{t}\left(s-p\right)-s-\left(1+\theta \right)f$$

for $f>\underline{f}$ (which by Proposition 3 holds in the optimum if θ is sufficiently small) and if there is no fee discrimination. Using *p *= (1 + *s *+ *v*(*s*))/2 from (7) and

$$2\tilde{t}=1-p+s=\frac{1+s-v\left(s\right)}{2}$$

from (1), this can be rewritten as

$$PW^{NFD}\left(f>\underline{f}\right)=Ū-1∕4-\frac{1+s-v\left(s\right)}{2}\frac{1-s+v\left(s\right)}{2}-s-\left(1+\theta \right)f.$$

Analogously, patient welfare under balance billing with fee discrimination (*FD*) is given by

$$\begin{array}{ll} PW^{FD}\left(f>\underline{f}\right) & =Ū-\frac{1}{4}-\frac{1+s\left(d\right)-2d-v\left(s\left(d\right)\right)}{2}\frac{1-s\left(d\right)+2d+v\left(s\left(d\right)\right)}{2}\;\\ & \;-s\left(d\right)-\left(1+\theta \right)\left[2\hat{t}\left(f+d\right)+2\tilde{t}\left(f-d\right)\right]\;\\ & =Ū-\frac{1}{4}-\frac{1+s\left(d\right)-2d-v\left(s\left(d\right)\right)}{2}\frac{1-s\left(d\right)+2d+v\left(s\left(d\right)\right)}{2}\;\\ & \;-s\left(d\right)-\left(1+\theta \right)\left[f+d\left(2d+v\left(s\left(d\right)\right)-s\left(d\right)\right)\right].\;\\ & \; \end{array}$$

Hence, the change in patient welfare Δ*PW *= *PW^FD ^*- *PW^NFD ^*induced by fee discrimination is given by (33).

### A.5 Proof of Proposition 5

The last term of equation

(A34) $$\frac{\text{d}\Delta PW}{\text{d}d}{|}_{d=0}=-\frac{\text{d}s}{\text{d}d}{|}_{d=0}+\frac{1-v^{\prime }\left(s\right)}{2}\frac{\text{d}s}{\text{d}d}{|}_{d=0}\left(s-v\left(s\right)\right)+\theta \left(s-v\left(s\right)\right).$$

θ(*s *- *v*(*s*)) measures the effect of *d *on the distortion costs. This term is unambiguously positive for *s *≠ 0 because *s *- *v*(*s*) *>*0 and increases with θ. Noting that the first two terms on the right hand side of (34) do not depend on θ, we can therefore conclude that a small amount of payer fee discrimination increases patient welfare if θ is sufficiently large, irrespective of the level of *f *chosen originally.

The first two terms in (34) account for two further effects of fee discrimination on patient welfare, namely the induced change in price and quality. As was shown above, if *s <*0, then a marginal increase in *d *leads to a higher price and a lower quality, which both lowers patient welfare. The sign of the first two terms in (34) is therefore negative in this case and counteracts the positive effect from the reduced distortion. By contrast, if *s >*0, we have d*s*/d*d <*0 by (31), meaning that the fee-only patients receive a higher quality. This has a positive impact on patient welfare. For *s >*0, the overall sign of the first two terms in (34) is therefore ambiguous in general since the price and quality effects work in opposite directions. However, we can show that the positive quality effect dominates if the fee is chosen so as to maximize patient welfare. Assuming an interior optimum, the first order condition is

(A.2) $$\frac{\text{d}PW^{NFD}}{\text{d}f}=\frac{1-v^{\prime }\left(s\right)}{2}\left(s-v\left(s\right)\right)\frac{\text{d}s}{\text{d}f}-\frac{\text{d}s}{\text{d}f}-\left(1+\theta \right)=0.$$

In addition, (31) implies

$${\left(\frac{\text{d}s}{\text{d}d}\right|}_{d=0}=\left(1-v^{\prime }\left(s\right)\right)\frac{\text{d}s}{\text{d}f}.$$

Substituting into (A.2) yields

$$\frac{1}{1-v^{\prime }\left(s\right)}\left(\frac{1-v^{\prime }\left(s\right)}{2}\left(s-v\left(s\right)\right){\left(\frac{\text{d}s}{\text{d}d}\right|}_{d=0}-{\left(\frac{\text{d}s}{\text{d}d}\right|}_{d=0}-\left(1-v^{\prime }\left(s\right)\right)\left(1+\theta \right)\right)=0$$

or,

$$-{\left(\frac{\text{d}s}{\text{d}d}\right|}_{d=0}+\frac{1-v^{\prime }\left(s\right)}{2}\left(s-v\left(s\right)\right){\left(\frac{\text{d}s}{\text{d}d}\right|}_{d=0}=\left(1-v^{\prime }\left(s\right)\right)\;\left(1+\theta \right)>0\;\text{if}\;s>0.$$

This confirms that if the fee is chosen optimally and *s >*0, then even the first two terms in (34) are positive in sum and hence a small amount of payer fee discrimination increases patient welfare irrespective of the size of θ.

## Endotes

**Endnote a**. McKnight provides a detailed history of the legislation on balance billing [7].

**Endnote b**. By assumption, there is no further price discrimination among price-paying patients.

**Endnote c**. For quality competition to be effective, the right-hand side of the equation must always be larger or equal than zero, hence we need $Ū\geq 1-{\tilde{t}}_{i}+s_{j}$.

**Endnote d**. This is a corrected version of the corresponding equation (9) in [1].

**Endnote e**. See p. 251 in [1].

**Endnote f**. See Section 2.4 in [1].

**Endnote g**. To derive the demand functions, we already needed the assumption that  exceeds a certain minimum value, which must be strengthened at this point.

**Endnote h**. Note that $\text{d}W^{p}∕\text{d}f\left(\underline{f}\right)=\left(v^{\prime }\left(\bar{s}\right)-1\right)\;\left(1∕2+v\left(\bar{s}\right)-\bar{s}\right)\;\text{d}s∕\text{d}f-\theta <0$ because $1+v\left(\bar{s}\right)-\bar{s}=0$ by (15). In addition, d*W^p^*/d*f *(f*) = -θ *<*0. Hence, by continuity of *W^p^*(*f*), if there is a maximum in $\left[\underline{f},f^{*}\right]$, there must also be a minimum to the left of the maximum satisfying (18).

**Endnote i**. Each physician makes a profit of π*^i ^*= 1/2.

**Endnote j**. See Section 5.3 for a further evaluation of fee discrimination.

**Endnote k**. By inspection of the first derivative ∂W*/∂*f *= (*v*'(*s*) - 1)(1/2 + *v*(*s*) - *s*)d*s*/d *f *there exists a local minimum for $\tilde{f}$ between $\underline{f}$ and f* satisfying $1∕2+v\left(s\left(\tilde{f}\right)\right)-s\left(\tilde{f}\right)=0$.

**Endnote l**. As mentioned in the proof of Proposition 1, this local maximum of the second-best welfare function (as well as the minimum) may not exist if θ is very high.

**Endnote m**. An alternative measure is the utility of the worst-off patient. It can be shown that under balance billing with $f>\underline{f}$ this is the person located at $\tilde{t}$ (see Figure 3). Otherwise the person at *t *= 1/2 is worst-off. For this welfare measure we obtain the same qualitative results.

**Endnote n**. For the critical value ${\hat{\theta }}_{ALL}$, we must have ${\hat{\theta }}_{ALL}\leq {\hat{\theta }}_{PW}$. This follows from equation (23): the condition for all patients to be better off is *h*(*f*, θ) *>*0. For an increase in patient welfare it is sufficient that *h*(*f*, θ)+(*s*(*f*) - *p*(*f*))^2 ^*>*0.

**Endnote o**. Note that in this case all patients must be better off if patient welfare is higher under balance billing: Under balance billing all patients pay price *p *= 1 + *c *for quality *s *= 0 and therefore obtain utility Ū-t-(1+c). Without balance billing utility from Medicare is $Ū-t-s^{o}-\left(1+\theta \right)f^{o}$ which is smaller than 1 + *c *if *PW^p^*(*f^p ^*= 0) *PW^o^*(*f^o^*) *>*0.

**Endnote p**. See the proposition on page 252 in [1]. Their claim is that efficiency rises if *d *is small and *f *close to the second-best level *f ^p ^*in their framework. However, in the proof, they refer to a situation where *s *approaches zero, which is associated with *f *= f*.
